# Mammary Stem Cells and Breast Cancer Stem Cells: Molecular Connections and Clinical Implications

**DOI:** 10.3390/biomedicines6020050

**Published:** 2018-05-04

**Authors:** Toni Celià-Terrassa

**Affiliations:** Cancer Research Program, IMIM (Hospital del Mar Medical Research Institute), 08003 Barcelona, Spain; acelia@imim.es; Tel.: +34-93-3016-1434

**Keywords:** mammary stem cells, cancer stem cells, cell plasticity, immune interplay, tumor microenvironment, targeting CSCs

## Abstract

Cancer arises from subpopulations of transformed cells with high tumor initiation and repopulation ability, known as cancer stem cells (CSCs), which share many similarities with their normal counterparts. In the mammary gland, several studies have shown common molecular regulators between adult mammary stem cells (MaSCs) and breast cancer stem cells (bCSCs). Cell plasticity and self-renewal are essential abilities for MaSCs to maintain tissue homeostasis and regenerate the gland after pregnancy. Intriguingly, these properties are similarly executed in breast cancer stem cells to drive tumor initiation, tumor heterogeneity and recurrence after chemotherapy. In addition, both stem cell phenotypes are strongly influenced by external signals from the microenvironment, immune cells and supportive specific niches. This review focuses on the intrinsic and extrinsic connections of MaSC and bCSCs with clinical implications for breast cancer progression and their possible therapeutic applications.

## 1. Introduction

The mammary gland epithelium is hierarchically organized and composed of two main cellular lineages: the basal/myoepithelial cells with contractile capacity, and the luminal cells with milk productive function [1,2]. Within this structure, the multipotent mammary stem cells (MaSCs) are at the top of the hierarchy, giving rise to progenitor cells and differentiated cells of both lineages [1,3,4]. Importantly, mammary gland organogenesis occurs in the adult organism after birth, and fully develops during puberty with the hormonal kick-off; therefore, MaSCs exist in the adult gland. The oestrus cycles and pregnancy lead to dramatic changes of the epithelium that require a pool of MaSCs with the ability to differentiate into the different lineages. In addition to the hormonal regulation of mammary gland development, the stroma and immune system also play a critical role in maintaining the MaSCs and restoring the homeostasis of the gland [1]. Indeed, the first evidence of adult MaSCs were obtained decades ago after the transplantation of segments of epithelium that regenerated the entire gland [5,6]. Years later, two studies using flow cytometry cell sorting followed by transplantation assays identified MaSC populations defined by Lin^−^CD24^+^/CD29^h^ and Lin^−^CD24^+^/CD49f^h^ and Sca-1^−^ [7,8]. These populations displayed myoepithelial features and multipotency to regenerate the entire functional gland after transplantation, therefore in such perturbed regenerative conditions, adult MaSCs are bipotent and have myoepithelial properties [9]. However, whether the adult MaSCs are bipotent or unipotent under physiological conditions is still a matter of controversy, since new lineage-tracing studies have demonstrated the existence of both unipotent MaSCs [10] and bipotent MaSCs [11]. Therefore, the current knowledge of the mammary gland contemplates the coexistence of bipotent and unipotent adult MaSCs [2]. 

In the breast malignancy, cancer stem cells (CSCs) can arise from: newly transformed MaSCs through the acquisition of genetic mutations; normal non-stem epithelial cells in which the self-renewal is acquired by oncogenic events and transformation; or differentiated cancer cells that dedifferentiate into a cancer stem cell-like phenotype, as a demonstration of cellular plasticity [12,13,14]. Importantly, breast cancer stem cells (bCSCs) were first isolated from human tumors and identified as Lin^−^CD24^−^CD44^+^ cells [15]. As with other human tissue CSCs, this population was functionally defined by the ability to initiate tumors in immunocompromised mice in vivo, self-renew across serial transplantations, and reproduce tumor heterogeneity deriving non-CSCs [16]. Like in the normal MaSC field, the perturbed physiological conditions of transplantation assays and the use of immunocompromised mice raised skepticism about the veracity of a distinctive population of CSCs. In addition, the relatively high abundance of tumor-initiating populations in certain cancer types, such as melanoma, indicated that these were not a rare special cell subpopulation [17]. However, lineage-tracing studies have robustly demonstrated the existence of CSCs in unperturbed primary tumors using spontaneous mouse models [18,19], including in breast cancer [20]. In the latter study, the authors used lineage-tracing intravital imaging and were able to observe the bCSC dynamics; this demonstrated that bCSCs can derive differentiated cells and that non-CSCs can become CSCs, proving the existence of cell plasticity [20], which has important clinical implications. In fact, there is special interest in studying CSCs because these populations are responsible for sustained tumor growth, drug resistance and relapse after chemotherapy [16]. Their abnormal self-renewal abilities also represent a major advantage for initiating tumor growth in distant sites, a process known as metastatic initiation [21]. Thus, a better understanding of mammary stem cell biology is essential to better understand the differences and weaknesses of bCSCs and unveil specific molecular targets with therapeutic applications to halt breast cancer progression. 

## 2. Connections between MaSCs and bCSCs

Mouse MaSC populations identified as Lin^−^CD24^+^CD29^h^ [7] and Lin^−^CD24^+^CD49f^h^ [8] expand during hyperplasia and neoplastic transformation in spontaneous breast cancer mouse models, such as MMTV-Wnt1 and MMTV-PyMT mice [7,22]. These populations are also enriched in *BRCA1* mutant tumorigenic cell lines and had increased tumorigenic ability in vivo [23], suggesting that the normal stem cell phenotype is leading hyperplasia and tumor initiation upon malignant oncogenic transformation. New studies have identified more defined normal MaSC markers, the Lgr5^+^ [11,24] and Procr^+^ [25] subpopulations repopulate the mammary gland with higher efficiency than previous markers. Both have been validated by transplantation assays and lineage tracing assays showing bipotency in the adult normal gland [11,25]. Interestingly, LGR5 and PROCR are also expressed in malignant CD44^+^ bCSC populations [26,27] and both take part in Wnt signaling, which is an important pathway in both, MaSCs and bCSCs [28,29]. Remarkably, Lgr5^+^ has been involved in promoting bCSC maintenance and breast cancer progression, and predicts poor overall patient survival [26,30]. In the human breast, normal MaSCs are defined as EpCAM^−/low^/CD49f^h^ by their functional ability to repopulate all lineages in the mammary gland [31]. This population of EpCAM^−/low^/CD49f^h^ human MaSCs also express typical markers of malignant bCSCs CD24^−^/CD44^+^ [32]. Interestingly, the presence of EpCAM^−/low^/CD49f^h^ in breast tumors is associated with poor clinical prognosis [33], indicating the overlap between normal and malignant stem cell markers in human disease. In addition, other human MaSCs, identified based on their ability to retain the PKH26 dye, have a similar profile of CD24^h^/CD49f^hi^/DNER^hi^/DLL1^hi^, which correlates with aggressiveness and poor prognosis of human breast cancer [34]. Another relevant marker of normal human MaSCs and malignant bCSCs is the ALDH+ activity in cell populations. The presence of this population in breast tumors is strongly associated with poor clinical outcome [35]. Overall, several markers have been described for MaSCs and bCSCs with high overlap between the normal and malignant stem cells, suggesting that these markers are faithful to the stem cell phenotypes and their properties, from normal tissue regeneration to cancer initiation. 

**Figure 1:**
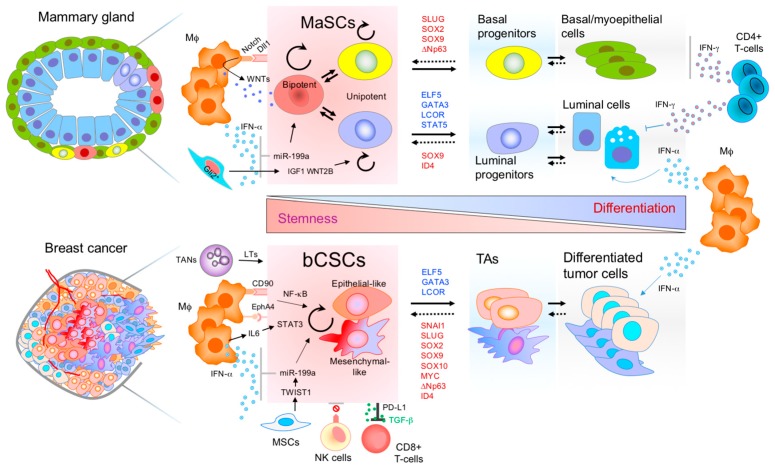
Molecular networks and immune regulation of mammary stem cells (MaSCs) and breast cancer stem cells (bCSCs). Similar immune cell components form the stem cell niche and the microenvironment of both stem cell phenotypes, normal and malignant. Transcription factors (TFs) inducing stem cell properties in the normal mammary gland (red letters), also induce stemness in breast cancer cells and increase tumor aggressiveness. On the other hand, luminal differentiation TFs of the mammary gland (blue letters), also induce differentiation in breast cancer cells and tumor suppression. Breast CSCs can be epithelial-like CSC or mesenchymal-like CSCs. The hierarchical organization of both tissues is highly dynamic, and the stem cell pool can be replenished from non-stem cell populations. Therefore, transitions can be bidirectional demonstrating cellular plasticity and environmental regulation of the mammary epithelial cells. Mφ, macrophage; TANs, tumor associated neutrophils; MSCs, mesenchymal stem cells; NK, natural killer; TAs, transit amplifying cells; LTs, leukotrienes.

Many molecular networks and cell fate regulators essential for cellular commitment and stemness are common between MaSCs and breast CSCs (Figure 1). This is proven by the similarities among their mRNA and miRNA transcriptomic profiles [32,36]. Moreover, critical pathways maintaining the stem cell phenotype are the same in normal MaSCs and breast CSCs. The main pathways shared are Hedgehog, Notch, JAK-STAT, NF-κB, and Wnt [37,38]. RANK/L activation also governs both MaSC and bCSC fate, inducing their expansion and tumorigenic potential [39,40,41]. The transcription factors directly regulating MaSC fate are similarly critical for the regulation of bCSCs (Figure 1). For instance, SLUG and SOX9 were shown to regulate MaSC activity in the mammary gland, as well as increase the tumorigenic and metastatic initiation ability of bCSCs [42]. SOX10 and the pluripotency factors MYC and SOX2 are implicated in the maintenance of MaSCs and bCSC phenotypes [43,44,45,46,47]. Interestingly, the combined expression of SOX9/SOX2 has been shown to be beneficial during metastatic latency for sustaining the survival of breast metastatic slow cycling CSCs [48]. Another MaSC transcription factor, the ∆Np63, increases the tumorigenic potential of basal-like tumors engaging Wnt signaling [29]. Similarly, ID4 also maintains the MaSCs pool preventing luminal commitment and it is expressed in basal-like tumors with poor prognosis [49]. STAT3 drives CSC tumorigenesis and relapse in different cancer types [50,51], and it is a downstream player of JAK-STAT signaling, which is important for both MaSC and bCSC maintenance [52,53,54]. Recently, miRNAs, such as miR-199a, have been show to promote MaSC activity and bCSCs in ER^−^ breast cancer, protecting them from differentiation elicited by environmental IFN-α [55]. Another study also detected miR-199a as one of the main miRNAs upregulated in human breast cancer patient CSCs [36]. MiR-31, upregulated by the RANKL/NF-κB pathway, fosters MaSC activity and tumorigenesis through direct repression of Wnt antagonists, which in turn favors the activation of Wnt signaling in MaSCs [56]. On the other hand, ELF5 and GATA3 are luminal differentiation transcription factors in the normal mammary gland, and induce bCSC differentiation, reducing their tumorigenic potential [57,58]. In addition, miR-200s also suppress stem cell properties by inducing luminal differentiation in the normal gland and reduce tumorigenesis by exhaustion of bCSCs [36]. MiR-30 and let-7 have also been implicated in reducing bCSC tumorigenesis [59,60]. Overall, many pluripotency transcription factors and cell fate regulators of stem cells are implicated in the regulation cancer stem cells, cell plasticity and tumor aggressiveness (Figure 1).

Another prominent network connection is through the activation of the epithelial-to-mesenchymal transition (EMT) that generates stem cell-like properties with CD24^−^/CD44^+^ markers, in both normal and malignant mammary cells [61,62]. This has been widely observed in a multitude of studies, and many of the EMT-inducing transcription factors also control MaSC and bCSC fates. Indeed, MaSCs resemble cells that undergo EMT based on morphological features, gene expression signatures, and activated pathways [1]. Furthermore, comparative molecular studies have demonstrated that gene expression profiles of MaSC populations overlap with Claudin-low breast cancer transcriptomic profiles, which harbor EMT-like properties and are enriched for CSCs [63,64,65]. In addition, this relationship suggests MaSCs as the origin of the Claudin-low/metaplastic breast cancer subtype [66,67]. However, under certain cellular contexts, EMT transcription factors do not induce stem cell properties, neither in normal nor cancer cells [68,69,70]. In fact, EMT attenuation is required to avoid extreme EMT that could lead to differentiation and loss of cell plasticity in breast cancer [71,72]. In particular, TWIST1 has been shown to control stemness independent of its function in inducing EMT [68]. Additionally, TWIST1 directly up-regulates miR-199a [73], which has been shown to induce breast cancer stem cell activity independently of EMT induction [55,74]. Therefore, downstream of the EMT-inducing TGF-β/TWIST1 signaling, the molecular mechanism engaging stem cell properties can be divergent of the mechanism inducing invasive properties. 

## 3. Mammary Stem Cells and Immune Interplay

The mammary gland stroma, and in particular the immune cells, play a critical role during the organogenesis of the gland [75]. Innate immune cells are important positive regulators of the mammary gland terminal end bud (TEB) elongation and branching [76]. Macrophages and eosinophils drive TEB invasion within the mammary fat pad environment [77] and mast cells help the branching process by releasing serine-proteases [78]. *Csf1^op/op^* mice, with homozygous *Csf1* mutation, have a severe reduction of macrophages. MaSC transplantation assays into *Csf1^op/op^* macrophage-depleted mammary fat pads showed compromised epithelial regeneration ability, demonstrating the macrophage supportive function for MaSCs [79]. Additionally, macrophage infiltration during mammary gland involution is critical for a proper clearing of dead epithelial cells in the involuting gland and should not affect the stem cell pool for future pregnancy cycles [80,81]. Macrophages fluctuate during mammary gland development, reaching higher levels during lactation-involution and tumorigenesis [55]. These mammary gland macrophages secrete IFN-α and mediate a differential effect on luminal progenitor/mature cells compared to the MaSCs. While MaSCs are protected from the IFN-α suppressive intrinsic effects (cell cycle arrest, apoptosis and differentiation), the luminal cells are highly sensitive to terminal differentiation upon IFN-α cellular response [55] (Figure 1). Therefore, the interplay with immune cells diverges between stem cells and differentiated cells, which is critical for mammary gland repopulation.

The adaptive immune system can also regulate luminal differentiation in the mammary gland (Figure 1). An elegant study showed that antigen-presenting cells activate CD4^+^ T helper 1 (Th1) cells, which release IFN-γ and suppresses mammary epithelial expansion and branching by negatively regulating luminal differentiation [82]. The authors showed that this effect was mediated by STAT1 activation in luminal cells, which has previously been reported to prevent luminal progenitor expansion [83], in contrast to other STATs involved in luminal differentiation, such as STAT5 and STAT6 [84]. Interestingly, this IFN-γ effect is distinct from the luminal differentiation mediated by IFN-α (IFN-type-I) mentioned above [55], however both studies showed that interferons impair the mammary gland epithelial development. Remarkably, in both studies the basal lineage cells are not sensitive to the interferon signaling response and only luminal cells are responsive. Further studies should provide new insights in such differential responses in basal and luminal cells.

While multiple studies have reported important roles of the immune cells and other stromal components in the mammary gland, the MaSC niche remained elusive until very recent findings. Gli2^+^ stromal cells have been shown to form a supportive niche for MaSCs, supplying them with essential factors IGF1 and WNT2B in response to estrogens and growth hormones [85]. Importantly, another very recent study has identified a macrophageal niche for MaSCs [86]. Dll1^+^ MaSCs directly interact with Notch in macrophages: this direct interaction triggers the expression of Wnt factors in macrophages, which are secreted and feedback MaSCs maintaining their setmness (Figure 1). This further demonstrates the critical interplay between immune cells and stem cells for the mammary gland development.

## 4. Breast Cancer Stem Cells and the Immune Microenvironment

As with normal MaSCs, macrophages are also very important players for bCSCs to maintain their stem-like state [87]. This study showed evidence of a direct macrophageal niche for breast CSCs, maintaining their stemness through heterotypic Thy1 (CD90) and EphA4 interactions and downstream induction of Src and NF-κB signaling (Figure 1). Indeed, NF-κB has been previously shown to be important for bCSC maintenance and the recruitment of macrophages in breast tumors [88]. Additionally, macrophages can secrete IL-6 which in turns activates STAT3, mediating self-renewal of breast cancer cells [89]. Moreover, as in normal MaSCs, ER^−^ bCSCs do not respond to the suppressive IFN-α from infiltrating macrophages [55]. Therefore, the evasion from immune-derived interferon is a common feature of normal and breast cancer stem cells (Figure 1). In breast cancer lung metastasis, neutrophils secrete leukotrienes that promote metastasis by expanding the CD24^+^CD90^+^ bCSC population in the MMTV-PyMT spontaneous mouse model. The same study also showed a similar expansion of the bCSC population in 4T1 cells and MDA-MB-231 cells [90]. During metastatic latency, slow-cycling bCSCs express SOX2 and SOX9 with a concomitant downregulation of ULBP ligand activators of natural killer (NK) cells and cell death-signal receptors [48]. This finding shows that breast cancer cells, by acquiring a stem cell-like phenotype, also acquire immune evasion abilities (Figure 1). Indeed, disseminated breast tumor cells express an EMT-like stem cell program while remaining dormant [91], and previous studies have shown that EMT induces immunosuppressive properties in breast cancer cells [92]. Therefore, this can be especially relevant when solitary disseminated tumor cells (DTCs) arrive at a distant site and need to be protected from the immune surveillance of the new hostile environment, while preserving their self-renewal ability until they are ready to form overt metastasis. On the other hand, CD8^+^ T-cells have been shown to promote EMT and increase the bCSC content in breast tumors [93], which is intriguing considering that EMT can engage immunosuppressive effects on T-cells. In general, more studies are required in this field to study the dynamics of bCSC-immune reciprocal interactions during breast cancer progression.

## 5. Clinical Implications of bCSCs and Cellular Plasticity

CSCs are characterized by their aggressiveness and chemoresistance/radioresistance. They are therefore considered the root of acquired resistance and relapse [38]. These populations are present in breast tumors and their content is strongly associated with poor metastasis-free survival and overall survival [94]. For instance, the aggressive triple-negative breast cancer (TNBC) subtype has high content of bCSCs [95]. Interestingly, within the breast tumor heterogeneity, distinct bCSC types are found: CD24^−^/CD44^+^; ALDH^+^; and CD24^−^/CD44^+^/ALDH^+^, all with different features and transcriptomic profiles [96]. These populations are independently present in the different breast cancer subtypes [32]; however, the proportion of them might vary between subtypes. For instance, ALDH^+^ bCSCs are more common of luminal and HER2 subtypes [97,98], and CD24^−^/CD44^+^ are enriched in basal-like and Claudin-low breast cancers [32,66]. Therefore, although different bCSCs may need to be targeted across all breast cancers, their preponderance in each particular breast cancer subtype should be considered for therapeutic strategies.

Cellular plasticity is defined as the interconversion of cellular phenotypes and degrees of differentiation/dedifferentiation [99]. The interconversion and cell plasticity of bCSCs is an important aspect to take into consideration during therapy. Remarkably, and without doubt, CSCs can arise from progenitor or tumor differentiated non-CSCs due to cellular plasticity (Figure 1). This may happen under dramatic microenvironmental circumstances or just by exhaustion of the CSC population [12,99]. Cell plasticity dynamics have been proven in bCSCs during tumor progression of the MMTV-PyMT model [20]. Moreover, the therapeutic approaches targeting bCSCs have shown that bCSCs can stochastically transition from non-CSCs to regenerate the CSC pool, affecting tumor resistance to paclitaxel and 5-fluorouracil [100]. These studies highlight the clinical relevance of the phenotypic interconversion as an additional mechanism of chemoresistance; consequently, both CSCs and non-CSCs must be targeted simultaneously to avoid interconversions into drug resistant phenotypes. A major mechanism of cellular plasticity is driven through the epithelial-to-mesenchymal/mesenchymal-to-epithelial bidirectional transitions (EMT/MET). These transitions generate both EMT-like (mesenchymal-like) CSCs and MET-like (epithelial-like) CSCs which maintain their stemness status but possess different properties that can provide different drug resistances [32]. Indeed, EMT is widely known to induce chemoresistance; hence, residual cells after hormonal therapy (letrozole) or chemotherapy with docetaxel display EMT-like CSC signatures and markers [101]. A 16,000-compound library screen in EMT-like CSCs found salinomycin as a potent drug targeting CSCs, reducing CSC content by 100-fold compared to paclitaxel [102]. Interestingly, as EMT has been shown to display a gradient of intermediate states [103], a recent study has quantified the differential drug resistance and sensitivity across epithelial-like and mesenchymal-like cancer cell states [104]. Accordingly, it is necessary to have a broad spectrum of applicable drugs for the range of phenotypes observed in each cancer type. Overall, the detailed depiction of cellular plasticity interconversions and EMT dynamic gradients will definitely help breast cancer treatments by informing combinatorial strategies.

## 6. New Therapeutic Opportunities Disrupting the Breast CSC–Immune Interplay

The molecular disruption of the communication between CSCs and immune cells offers a great opportunity to specifically target CSCs. An obvious strategy is to break their connection with the supportive niche; for instance, blockading the heterotypic interaction with the macrophageal niche [87], either by blocking CD90 or EpHA4, would halt bCSC maintenance. It is also possible to block macrophage recruitment and polarization with anti-IL-6 (Siltuximab), anti-CCL2 or anti-CSF-1, to avoid their interplay with bCSCs [105]. The Alox5 inhibitor zileuton (Zil) can block the leukotriene secretion of neutrophils and avoid the expansion of metastatic bCSCs [90]. On the other hand, bCSCs and especially those in an EMT-like state can secrete high levels of TGF-β in the tumor microenvironment [106], which mediates the immunosuppression of CD8^+^ T-cell activity nearby and shields them from T-cell infiltration [107]. In fact, a very recent study in colorectal cancer has demonstrated that the administration of TGF-β inhibitor eliminates TGF-β-mediated immunosuppressive effects on the immune system, thereby enhancing immunotherapy efficiency [108]. In addition, EMT-like breast cancer cells express more PD-L1 and fewer MHCs, indicating the need to design specific immunotherapeutic approaches for these cells [92,109] (Figure 1). Notably, CSC-vaccines represent another interesting therapeutic strategy to specifically target CSCs. Dendritic cell (DC) vaccines are generated, exposing DCs to CSCs lysates that uptake and process CSC specific antigens to present to T-cells. Therefore, the administration of DC-vaccines mediate immune responses specifically recognizing CSC antigens [110]. In addition, another interesting strategy utilizes oncolytic viruses, which have been shown to specifically target CSCs. As shown before, bCSCs have a defective IFN-α response [55], thus their ability to activate an appropriate antiviral response is limited, and thus the oncolytic virus can specifically kill these cells [111,112,113]. Future studies should investigate possible synergistic effects of a combination of these treatments with immunotherapy and/or conventional therapy.

## 7. Conclusions

The identification and comprehension of MaSC biology has tremendously impacted our knowledge of breast CSCs. During the last decade, new technologies have allowed for the identification of adult MaSCs, the existence of bCSCs, and proven cellular plasticity. However, we still need to improve the identification of the main niches and pure bCSC populations. In some cases, different markers define distinct bCSCs; however, the combination of these markers identify overlapping rare bCSC with higher tumorigenic ability [32]. This suggests that the combination of different bCSCs markers is identifying a cleaner population of bCSCs, therefore indicating that current markers are still far from exclusively marking bCSCs, and are likely to overlap with non-CSCs, which masks the downstream biological and clinical studies of bCSCs. Future approaches should focus on the identification of functionally-related CSC makers that faithfully reflect the stem cell phenotype. Advanced technologies, in particular single cell-RNA sequencing, are powerful tools to identify unprecedented markers that recapitulate pure bCSCs as a reflection of their stem cell abilities. Additionally, as stated in this review, the properties and molecular networks governing MaSCs and bCSCs share many similarities, which illustrates the importance of studying stem cell biology in cancer. Thus, it is critical to continue studying their similarities and differences in order to unveil specific bCSC distinctions for new efficient specific therapies. Many intrinsic molecular and extrinsic environmental differences are already well differentiated between both systems, and well-designed lineage-tracing studies precisely show different dependencies on transcriptions factors: for example, that SNAI1 is more critical for bCSCs and SLUG for MaSCs [62] (Figure 1). Moreover, new 4D imaging technologies should be able to report the precise dynamics of bCSC plasticity at different moments of mammary gland development and carcinogenesis. This will provide essential knowledge to avoid chemoresistance phenomena due to phenotypic interconversion. Parallel advances in both fields will spearhead essential knowledge in breast cancer biology and the design of new therapeutic approaches.
